# The Association between Eating-Out Rate and BMI in Korea

**DOI:** 10.3390/ijerph16173186

**Published:** 2019-08-31

**Authors:** Hwi Jun Kim, So Yeon Oh, Dong-Woo Choi, Eun-Cheol Park

**Affiliations:** 1 Department of Public Health, Graduate School, Yonsei University, Seoul 03722, Korea; 2 Institute of Health Services Research, Yonsei University, Seoul 03722, Korea; 3 Department of Preventive Medicine, Yonsei University College of Medicine, Seoul 03722, Korea

**Keywords:** BMI, obesity, overweight, eating-out rate, Korea National Health and Nutrition Examination Survey

## Abstract

Previous research suggests that adult men consume larger amounts of calories while eating-out than when eating meals prepared at home. Therefore, this study aimed to investigate the association between the daily eating-out rate and body mass index (BMI) in the Korean population. The study used data from 18,019 individuals aged ≥19 years who participated in the Korea National Health and Nutrition Examination Survey (KNHANES) from 2013 to 2016. BMI was measured according to the Asia-Pacific BMI measurement criteria. A multinomial logistic regression analysis was used to examine the validity of the association between the eating-out rate and BMI. In this population, women with higher eating-out rates were found to have higher BMIs. Specifically, the risks of becoming obese or overweight increased among those with a 1%–50% (obesity odds ratio (OR) = 1.28, 95% confidence interval [CI]: 1.09–1.51; overweight OR = 1.38, 95% CI: 1.14–1.64) or 51%–100% daily eating-out rate (obesity OR = 1.51, 95% CI: 1.24–1.84; overweight OR = 1.50, 95% CI: 1.20–1.87), relative to those who reported never eating-out. By contrast, no statistically significant association between the daily eating-out rate and BMI was observed among men. Notably, we observed positive associations of the daily eating-out rate with obesity and being overweight in South Korean women, but not men. Our findings suggest that education about proper habits when eating-out is needed to prevent obesity.

## 1. Introduction

According to a survey conducted by the World Health Organization (WHO), the proportion of obese individuals worldwide has increased by approximately three-fold from 1975 to 2016 [1]. Although obesity is a non-communicable disease, the ever-increasing population of affected individuals has become a global issue [2,3,4], particularly as the WHO has reported that the health consequences of obesity, particularly chronic diseases and death, are as deleterious as those of smoking [5]. Consistent with global trends, the rate of obesity is steadily increasing in South Korea. The 2016 version of the Korea National Health and Nutrition Examination Survey (KNHANES) determined an obesity rate of approximately 30% [6], and predictions suggest that the social problems caused by obesity will soon become extreme. Although obesity does not have specific symptoms, it has been correlated with cardiovascular disease, hypertension, type II diabetes, musculoskeletal disorders, and some types of cancer [7,8,9,10,11]. Therefore, it is equally important to prevent and treat/manage obesity [12,13,14].

Modern occupational shifts to more knowledge-based production industries [15] have led to relative decreases in opportunities for physical activity. This is certainly true in South Korea, where much of the working population remains seated during working hours [16]. Recent years have also seen an increase in the amount of appetite-stimulating media, such as cooking shows and restaurant reviews. Simultaneously, an increasing number of people show they prefer to eat out than at home. In Korea, this phenomenon has been demonstrated by increases in both the frequency of eating-out and the decreased proportion of household expenditure on food purchases. For example, the frequency of eating-out increased from 23.6% in 1990 to 45.1% in 2015 in Korea, while the proportion of household expenditure on food decreased from 76.4% to 54.9% [8,9,17].

According to a previous study of the lunchtime consumption habits of adult men, greater amounts of calories were consumed when eating-out than when eating lunch at home [18]. This led us to speculate on a potential connection between the increased frequency of eating-out and the increasing incidence of overweightness/obesity in Korea, using the body mass index (BMI) as an indicator. Such a correlation might be a main factor affecting the incidence and prevention of obesity.

We expected this phenomenon to be observed equally due to the increasingly Western-style Korean eating-out culture. Therefore, this study was conducted to investigate the relationship between daily eating-out rate and BMI among Koreans.

## 2. Materials and Methods

### 2.1. Data Collection and Study Participants

The KNHANES is a cross-sectional survey of a nationally representative, all-age population conducted annually by the Korea Centers for Disease Control and Prevention (KCDC) since 1998. From among the 31,098 respondents to the 2013–2016 KNHANES, we selected only adult respondents aged 19 years or older (*n* = 24,095).

We used two-stage filtering to extract participants who responded to a question related to the frequency of eating-out. First, we classified participants as obese, overweight, underweight, or normal weight according to the BMI classification (*n =* 22,883). Second, the average number of meals per day was calculated as the average of number of breakfasts, lunches, and dinners consumed, and the daily frequency of eating-out was stratified as “more than twice per day,” “less than once per day (1 to 24 times per month)*,”* and “none (less than once per month)*.”*

The frequency of daily meals may vary from person to person. We focused on eating-out rates for a more detailed analysis. Therefore, we calculated the daily eating-out rate as the frequency of eating-out per day/daily food intake rate per day (*n =* 19,704). Additional details are provided in the next section.

We excluded participants for whom the following data were missing: Marital status (*n =* 31), household income (*n =* 92), educational level (*n =* 1482), occupation (*n =* 29), and physical activity (*n =* 51). Therefore, a final sample population of 18,019 people was included in our study analysis.

The Korea National Health and Nutrition Examination Survey (KNHANES), which we used, corresponds to the research conducted by the government for public welfare in accordance with the Korea Bioethics Act. Therefore, it was possible to conduct this investigation without consideration of the research ethics review committee.

### 2.2. Variables

Our independent variable was the daily rate of eating-out. Because the KNHANES did not include variables that could be used to calculate this rate, we generated an average rate of eating-out. First, the average number of meals per day was determined from responses to the question, “How many times a week did you eat breakfast (lunch, dinner) in the last year?” The possible answers were 5–7, 3 or 4, 1 or 2, or no times per week. Subsequently, we divided the results by 7 to calculate the average number of meals. Through those three indicators, we divided into groups, those that had meals once a day, a group that eats twice a day, and a group that eats more than three times a day. Second, the question used to determine the frequency of eating-out and set the variable of interest was, “On average, during the past year, how often did you eat out rather than home-cooked food?” This item had 7 possible responses: More than twice per day, once per day, 5 or 6 times per week, 3 or 4 times per week, 1 or 2 times per week, 1–3 times per month, and never (less than once per month). Because the answers to this question differed, we sorted the respondents using the following reset criteria: more than twice per day, less than once per day (once per day, 5–6 times per week, three to four times per week, 1 to 2 times per week, 1–3 times per month), and never (less than once per month). The daily rate of eating-out was calculated using the following formula: Daily eating-out frequency/daily eating frequency × 100.

The BMI status was set as the dependent variable. We generated these data based on the following Asia-Pacific BMI standards: Normal, 18.5–22.9 kg/m^2^; underweight, <18.5 kg/m^2^; overweight, 23–24.9 kg/m^2^; and obese, ≥25 kg/m^2^.

### 2.3. Covariates

The sociodemographic factors of sex (men, women), age (20–29, 30–39, 40–49, 50–59, 60–69, ≥70 years), and the socioeconomic factors of marital status (unmarried, married, and once married (divorced, separated, or widowed)), monthly household income (high, medium, medium-high, and medium-low, low), educational level (elementary school or less, middle school, high school, and college or higher), and occupation (workers and non-workers) were included. Health-related behaviors were categorized as follows: Smoking status (yes, no), alcohol status (yes, no). Daily energy intake was determined using the recommendations for energy intake by sex and age published by the Ministry of Health and Welfare in 2015 [19]. According to Korean energy intake standards, the recommended energy intake varied according to age, sex, and pregnancy. Therefore, we calculated the recommended nutrient intake to be in the range of ±100 kcal to determine whether the nutrient intake was appropriate or not. Physical activity to the ACSM guideline was determined using data regarding the frequency of physical activity participation. The time, frequency, and intensity of exercise were taken into consideration to conduct a more detailed analysis of the physically active (vigorous physical activity once per week for 20 min or more, 3 days a week or more, or moderate physical activity or walking exercise performed once for more than 30 min per week for 3 days or more) and non-activite groups [20]. Finally, diabetes is closely related to obesity [21,22]. Therefore, we added diabetes as a covariate. Diabetes-related items were stratified by the clinical (i.e., physician’s) diagnostic or non-diagnostic status.

### 2.4. Statistical Analysis

The chi-square test was used to examine significant differences in BMI depending on the eating-out rate. We also used multinomial logistic regression because we had four dependent variables (obesity, overweight, underweight, and normal weight). Multinomial logistic regression is used when the dependent variable in question is nominal (equivalently categorical, meaning that it falls into any one of a set of categories that cannot be ordered in any meaningful way) and for which there are more than two categories. That method is a particular solution to classification problems that use a linear combination of the observed features and some problem-specific parameters to estimate the probability of each particular value of the dependent variable. Multinomial logistic regression analysis was used to determine odds ratios (ORs) and 95% confidence intervals (CIs) after adjusting for covariates. Additionally, subgroup analyses according to the eating-out rate and BMI were conducted. For all data analysis, we used SAS version 9.4 (SAS Institute, Cary, NC, USA) and the significance level was set at *p*-value < 0.05.

## 3. Results

### 3.1. Study Participants

Table 1 lists the general characteristics of the study population, stratified by sex. Approximately 90% of Koreans reported some frequency of eating-out. On the other hand, 517 men (7.2%) and 1147 women (10.6%) answered that they do not eat out at all. Among the respondents who said that they eat out, 5061 men (70.1%) and 7,658 women (71.0%) were included in the eating-out rate of 1%–50%. Within the category of a 51%–100% rate of eating-out, 1657 (22.8%) were men and 1989 (18.4%) were women. Although similar proportions of men and women comprised the 1%–50% group, the distribution in the 51%–100% group indicated a greater exposure to eating-out among men than among women. Among male participants, 2769 (38.3%), 1878 (25.9%), 208 (2.81%), and 2375 (32.9%) met the BMI criteria for obesity, overweight, underweight, and normal weight, respectively. Among female participants, 3230 (29.9%), 2327 (21.5%), 525 (4.9%), 4,712 (43.7%) met the BMI criteria for obesity, overweight, underweight, and normal weight, respectively. Among the subgroup of women who reported never eating-out, 37.8%, 22.5%, 3.6%, and 36.1% met the criteria for obesity, overweight, underweight, and normal weight, respectively. Among the subgroup of men who reported never eating-out, 29%, 25.5%, 7.5%, and 37.9% met the criteria for obesity, overweight, underweight, and normal weight, respectively. For men with a rate of eating-out of 1%–50%, the corresponding values were 1927 (38%), 1332 (26.3%), 117 (2.3%), and 1685 (33.3%), respectively, while among those with a rate of eating-out of 51%–100%, the corresponding values were 692 (42.0%), 414 (25.0%), 47 (2.9%), and 494 (30.0%), respectively. For women with a rate of eating-out of 1%–50%, the corresponding values were 2,260 (29.5%), 1686 (22.0%), 352 (4.6%), 3360 (43.9%), respectively, while among those with a rate of eating-out of 51%–100%, the corresponding values were 536 (27.0%), 383 (19.3%), 132 (6.6%), 938 (47.2%), respectively. In summary, approximately 60% of the men in each group exceeded the normal weight category, regardless of their rate of eating-out. Approximately 50% of the women in each group exceeded the normal weight category, regardless of their rate of eating-out.

### 3.2. Factors Associated with Eating-out Rate and BMI

Table 2 presents the results of factors associated with the frequency of eating-out and the BMI. A multinomial logistic regression analysis corrected for covariance revealed a significant correlation between the eating-out rate and BMI only among women. In this group, the likelihood of becoming obese or overweight was higher among those with a daily eating-out rate of 1%–50% (obesity OR = 1.28, 95% CI: 1.09–1.51; overweight OR = 1.38, 95% CI: 1.14–1.64) or 51%–100% (obesity OR = 1.51, 95% CI: 1.24–1.84; overweight OR = 1.50, 95% CI: 1.20–1.87) relative to those who reported never eating-out. It is also analyzed that people with diabetes are more likely to be obese (men’s obesity OR = 1.53, 95% CI: 1.27–1.84; women’s obesity OR = 1.65, 95% CI: 1.39–1.96).

### 3.3. Association between BMI and Eating-out Rate by Socioeconomic Status

Table 3 presents the results of a subgroup analysis stratified by socioeconomic status. Among men, those who were married and had a daily eating-out rate of 51%–100% were 1.44 times more likely to be obese. Among women, those who were married and had a daily eating-out rate of 1%–50% were 1.30 times more likely to be obese. Furthermore, those with a low household income and those with an education level of less than elementary school were 1.34 and 1.27 times more likely to be obese, respectively. In the analysis of occupation, the probability of obesity for women with and without occupation increased by 1.38 times and 1.24 times, respectively, regardless of occupation. Among those with a daily eating-out rate of 51%–100%, married women were 1.56 times more likely to become obese, and those with a low household income were 1.49 times more likely to be obese. In the analysis of occupation, the probability of obesity for women with and without occupations increased by 1.44 times and 1.67 times.

## 4. Discussion

Previous studies conducted in other countries identified a positive association between the frequency of eating-out with BMI [23,24,25,26,27,28,29,30]. Specifically, previous studies conducted in Europe suggested that foods consumed while eating-out tend to contain higher amounts of energy-dense macronutrients, such as fat and sugar, compared to those prepared at home [24], while a Brazilian study found a positive correlation between the frequency of food intake with adult weight gain in South America [23]. Our finding of a significant association between the eating-out rate and BMI among Korean adult women but not men was partially consistent with those previous reports. Our observations indicate that a high frequency of eating-out may correlate with a higher BMI among women. Particularly, we found that many married women who eat out frequently are overweight or obese. Furthermore, it was confirmed that women with frequent eating-out patterns were more likely to be obese, regardless of their occupational status. In addition, as in the previous study, the analysis of our study showed that a person with diabetes is likely to have a high BMI [22].

Our findings suggest a need for improvements in education, publicity, and policies regarding healthy eating habits, and indicate that institutional measures should be formulated to establish a healthy culture of eating-out. Approximately 92.8% of Korean men and 89% of Korean women have reported exposure to eating-out [31]. As the proportion of people who eat out has been on the rise lately, the government needs to review the various laws and regulations related to eating-out and lead a proper eating-out culture [32]. Such as, by providing eating-out guidelines for diabetics, providing calorie information for each food, and guidelines for using ingredients for restaurants. But before we do that, the relationship between the eating-out rate and obesity in women requires verification. According to a previous study conducted in the United States, the analysis of eating-out and BMI of premenopausal women showed higher BMIs in women who frequently eat out [28]. As with this prior study, according to our results, the more often Korean women eat out, the more they are affected in terms of their BMI. However, no significant results were found in men, only identifying the tendency that BMI may increase as more people eat out. This is in contrast to previous studies that suggested that BMI may increase with the rate of eating-out for both men and women [23,33,34]. To validate our findings, further investigations are recommended to see if there is a causal relationship between BMI and eating-out. Based on our research, causality in further studies will help to come up with measures to reduce obesity in certain populations.

Our research has numerous limitations. First, we used cross-sectional data, thus could not include accurate information about the food consumed during meals. To objectively determine the relationship between the eating-out rate and BMI, it would be necessary to compare calories from identical menus of foods prepared at home and in a restaurant. Second, data concerning the daily eating-out rate were derived from a combination of four indicators and was calculated as the ratio of numeric responses to the questions, “How often did you eat out on average during the past year?” and “How many times a week did you have breakfast (lunch, dinner) in the last year,” and multiplied by 100 to yield a percentage. Therefore, the measurement values may have been more unstable than values derived using a single index. Third, the relevant KNHANES question inquired about the average during the previous 1-year period. The response relies on the potentially incomplete and inaccurate memory of the respondent, thus may be subject to recall bias. Fourth, it is difficult to define obesity and overweightness according to the BMI standard, or to assume exposure to other diseases. For example, BMI measurements often suggest that people with an above-average muscle mass are overweight or obese.

Despite these limitation, our research has several strengths of note. First, the use of nationally representative data will allow our results to be generalized to the general adult population of South Korea. Second, as the statistical analysis was based on data collected over four consecutive years, the correlation between the rate of eating-out and BMI among Korean adults is relatively reliable. Third, our findings are consistent with previous studies suggesting that eating-out, compared to eating at home, may be associated with a higher caloric intake [23,24,25,26,35] and is more likely to increase BMI [36,37].

## 5. Conclusions

Our study found that the higher the rate of eating-out among Korean women, the higher their BMI. However, there were no similar associations observed among men. Our research will help the Korean Government and organizations to review and improve their regulations and implement them, to create a healthy and correct eating-out culture for the people.

## Figures and Tables

**Table 1:** General characteristics of study observations (2013–2016).

Variable	Men										*p*-Value	Women										*p*-Value
	Total		Obesity		Overweight		Underweight		Normal			Total		Obesity		Overweight		Underweight		Normal		
	N	(%)	N	(%)	N	(%)	N	(%)	N	(%)		N	(%)	N	(%)	N	(%)	N	(%)	N	(%)	
**Daily eating-out rate**											<0.0001											<0.0001
None	517	7.2	150	29.0	132	25.5	39	7.5	196	37.9		1147	10.6	434	37.8	258	22.5	41	3.6	414	36.1	
1%–50%	5061	70.1	1927	38.1	1332	26.3	117	2.3	1685	33.3		7658	71.0	2260	29.5	1686	22.0	352	4.6	3360	43.9	
51%–100%	1647	22.8	692	42.0	414	25.1	47	2.9	494	30.0		1989	18.4	536	27.0	383	19.3	132	6.6	938	47.2	
**Age**											<0.0001											<0.0001
20–29	777	10.8	260	33.5	159	20.5	35	4.5	323	41.6		1071	9.9	161	15.0	133	12.4	155	14.5	622	58.1	
30–39	1065	14.7	500	47.0	258	24.2	20	1.9	287	27.0		1892	17.5	380	20.1	294	15.5	155	8.2	1063	56.2	
40–49	1262	17.5	550	43.6	332	26.3	18	1.4	362	28.7		2023	18.7	516	25.5	432	21.4	90	4.5	985	48.7	
50–59	1335	18.5	556	41.7	384	28.8	28	2.1	367	27.5		2127	19.7	708	33.3	520	24.5	43	2.0	856	40.2	
60–69	1437	19.9	524	36.5	395	27.5	31	2.2	487	33.9		1827	16.9	733	40.1	489	26.8	25	1.4	580	31.8	
≥70	1349	18.7	379	28.1	350	26.0	71	5.3	549	40.7		1854	17.2	732	39.5	459	24.8	57	3.1	606	32.7	
**Marital status**											<0.0001											<0.0001
Unmarried	1180	16.3	437	37.0	246	20.9	47	4.0	450	38.1		1186	11.0	185	15.6	139	11.7	171	14.4	691	58.3	
Married	5538	76.7	2155	38.9	1499	27.1	132	2.4	1,752	31.6		7477	69.3	2230	29.8	1673	22.4	289	3.9	3285	43.9	
Once married (divorced, separated, bereavement)	507	7.0	177	34.9	133	26.2	24	4.7	173	34.1		2131	19.7	815	38.2	515	24.2	65	3.1	736	34.5	
**Household income**											<0.0001											<0.0001
Low	1357	18.8	438	32.3	335	24.7	67	4.9	517	38.1		2270	21.0	886	39.0	551	24.3	73	3.2	760	33.5	
Medium-Low	1824	25.3	693	38.0	451	24.7	56	3.1	624	34.2		2710	25.1	906	33.4	593	21.9	126	4.7	1085	40.0	
Medium-High	1974	27.3	811	41.1	502	25.4	50	2.5	611	31.0		2863	26.5	804	28.1	575	20.1	137	4.8	1347	47.1	
High	2070	28.7	827	40.0	590	28.5	30	1.5	623	30.1		2951	27.3	634	21.5	608	20.6	189	6.4	1520	51.5	
**Educational level**											<0.0001											<0.0001
Elementary school	1325	18.3	422	31.9	346	26.1	64	4.8	493	37.2		3087	28.6	1334	43.2	785	25.4	69	2.2	899	29.1	
Middle school	823	11.4	297	36.1	225	27.3	28	3.4	273	33.2		1116	10.3	415	37.2	278	24.9	20	1.8	403	36.1	
High school	2477	34.3	966	39.0	604	24.4	63	2.5	844	34.1		3309	30.7	888	26.8	715	21.6	166	5.0	1540	46.5	
College or more	2600	36.0	1084	41.7	703	27.0	48	1.9	765	29.4		3282	30.4	593	18.1	549	16.7	270	8.2	1870	57.0	
**Occupation**											<0.0001											<0.0001
Workers	5117	70.8	2085	40.8	1347	26.3	103	2.0	1582	30.9		5179	48.0	1440	27.8	1096	21.2	267	5.2	2376	45.9	
Non-workers	2108	29.2	684	32.5	531	25.2	100	4.7	793	37.6		5615	52.0	1790	31.9	1231	21.9	258	4.6	2336	41.6	
**Smoking**											0.0061											0.0055
Yes	2230	30.9	866	38.8	528	23.7	76	3.4	760	34.1		360	3.3	120	33.3	63	17.5	29	8.1	148	41.1	
No	4995	69.1	1903	38.1	1350	27.0	127	2.5	1615	32.3		10,434	96.7	3110	29.8	2264	21.7	496	4.8	4564	43.7	
**Alcohol consumption**											0.0010											<0.0001
Yes	5046	69.8	1975	39.1	1331	26.4	122	2.4	1618	32.1		4172	38.7	1144	27.4	780	18.7	207	5.0	2041	48.9	
No	2179	30.2	794	36.4	547	25.1	81	3.7	757	34.7		6622	61.4	2086	31.5	1547	23.4	318	4.8	2671	40.3	
**Daily energy intake**											0.0005											0.7907
Less than recommended energy intake per day	3055	42.3	1144	37.5	770	25.2	110	3.6	1,031	33.8		5369	49.7	1624	30.3	1163	21.7	271	5.1	2311	43.0	
Recommended energy intake per day	1373	19.0	496	36.1	373	27.2	39	2.8	465	33.9		2456	22.8	732	29.8	516	21.0	110	4.5	1098	44.7	
More than recommended energy intake per day	2797	38.7	1129	40.4	735	26.3	54	1.9	879	31.4		2969	27.5	874	29.4	648	21.8	144	4.9	1303	43.9	
**Physical activity**											0.0167											0.0012
Yes	3630	50.2	1390	38.3	961	26.5	80	2.2	1199	33.0		4697	43.5	1335	28.4	1002	21.3	214	4.6	2146	45.7	
No	3595	49.8	1379	38.4	917	25.5	123	3.4	1176	32.7		6097	56.5	1895	31.1	1325	21.7	311	5.1	2566	42.1	
**Diabetes mellitus**											0.0114											<0.0001
No	6415	88.8	2429	37.9	1664	25.9	191	3.0	2131	33.2		9871	91.5	2795	28.3	2104	21.3	515	5.2	4457	45.2	
Yes	810	11.2	340	42.0	214	26.4	12	1.5	244	30.1		923	8.6	435	47.1	223	24.2	10	1.1	255	27.6	
**Total**	7225	100.0	2769	38.3	1878	26.0	203	2.8	2375	32.9		10,794	100.0	3230	29.9	2327	21.6	525	4.9	4712	43.7	

**Table 2:** Factors associated with eating-out rate and BMI (2013–2016).

	Body Mass Index																							
	Men												Women											
	Obesity				Overweight				Underweight				Obesity				Overweight				Underweight			
Variables	OR	95% CI			OR	95% CI			OR	95% CI			OR	95% CI			OR	95% CI			OR	95% CI		
**Daily eating-out rate**																								
None	1.00	-		-	1.00	-		-	1.00	-		-	1.00	-		-	1.00	-		-	1.00	-		-
1%–50%	1.09	(0.85)	-	(1.38)	0.98	(0.76)	-	(1.26)	0.53	(0.34)	-	(0.82)	1.28	(1.09)	-	(1.51)	1.36	(1.14)	-	(1.64)	0.81	(0.54)	-	(1.20)
51%–100%	1.25	(0.95)	-	(1.64)	1.10	(0.82)	-	(1.46)	0.71	(0.41)	-	(1.22)	1.51	(1.24)	-	(1.84)	1.50	(1.20)	-	(1.87)	0.77	(0.50)	-	(1.20)
**Age**																								
20–29	1.00	-		-	1.00	-		-	1.00	-		-	1.00	-		-	1.00	-		-	1.00	-		-
30–39	1.85	(1.41)	-	(2.43)	1.46	(1.06)	-	(2.01)	0.77	(0.38)	-	(1.55)	1.28	(0.99)	-	(1.67)	1.04	(0.78)	-	(1.39)	0.78	(0.57)	-	(1.08)
40–49	1.59	(1.19)	-	(2.12)	1.44	(1.03)	-	(2.01)	0.58	(0.27)	-	(1.26)	1.68	(1.28)	-	(2.20)	1.48	(1.10)	-	(1.98)	0.53	(0.36)	-	(0.77)
50–59	1.58	(1.17)	-	(2.13)	1.61	(1.14)	-	(2.28)	0.85	(0.39)	-	(1.83)	1.96	(1.49)	-	(2.60)	1.70	(1.26)	-	(2.30)	0.29	(0.18)	-	(0.46)
60–69	1.12	(0.82)	-	(1.53)	1.26	(0.88)	-	(1.79)	0.60	(0.27)	-	(1.37)	2.10	(1.56)	-	(2.82)	1.98	(1.44)	-	(2.72)	0.23	(0.13)	-	(0.41)
≥70	0.76	(0.54)	-	(1.06)	1.03	(0.71)	-	(1.50)	0.93	(0.41)	-	(2.13)	1.64	(1.20)	-	(2.26)	1.63	(1.15)	-	(2.29)	0.44	(0.24)	-	(0.79)
**Marital status**																								
Unmarried	1.00	-		-	1.00	-		-	1.00	-		-	1.00	-		-	1.00	-		-	1.00	-		-
Married	1.20	(0.95)	-	(1.51)	1.35	(1.03)	-	(1.76)	0.81	(0.44)	-	(1.50)	1.27	(1.00)	-	(1.62)	1.52	(1.17)	-	(1.99)	0.57	(0.42)	-	(0.78)
Once married (divorced, separated, bereavement)	1.21	(0.89)	-	(1.66)	1.39	(0.98)	-	(1.98)	1.07	(0.51)	-	(2.26)	1.17	(0.89)	-	(1.53)	1.40	(1.04)	-	(1.90)	0.72	(0.46)	-	(1.13)
**Household income**																								
Low	1.00	-		-	1.00	-		-	1.00	-		-	1.00	-		-	1.00	-		-	1.00	-		-
Medium-Low	1.03	(0.86)	-	(1.23)	1.02	(0.84)	-	(1.25)	1.02	(0.68)	-	(1.54)	1.02	(0.88)	-	(1.18)	0.98	(0.83)	-	(1.15)	1.07	(0.77)	-	(1.51)
Medium-High	1.11	(0.92)	-	(1.35)	1.12	(0.91)	-	(1.38)	1.06	(0.67)	-	(1.66)	0.90	(0.77)	-	(1.05)	0.90	(0.75)	-	(1.06)	0.88	(0.62)	-	(1.25)
High	1.05	(0.86)	-	(1.29)	1.22	(0.99)	-	(1.52)	0.67	(0.39)	-	(1.14)	0.70	(0.59)	-	(0.82)	0.87	(0.73)	-	(1.04)	1.18	(0.83)	-	(1.67)
**Educational level**																								
Elementary school	1.00	-		-	1.00	-		-	1.00	-		-	1.00	-		-	1.00	-		-	1.00	-		-
Middle school	0.90	(0.73)	-	(1.10)	0.94	(0.75)	-	(1.17)	1.28	(0.77)	-	(2.14)	0.68	(0.57)	-	(0.81)	0.79	(0.65)	-	(0.96)	0.76	(0.44)	-	(1.34)
High school	0.95	(0.77)	-	(1.18)	0.99	(0.79)	-	(1.25)	1.23	(0.71)	-	(2.14)	0.48	(0.41)	-	(0.57)	0.67	(0.56)	-	(0.80)	0.98	(0.62)	-	(1.56)
College or more	0.96	(0.83)	-	(1.10)	0.90	(0.77)	-	(1.06)	0.90	(0.60)	-	(1.36)	0.32	(0.26)	-	(0.38)	0.50	(0.40)	-	(0.61)	1.10	(0.68)	-	(1.77)
**Occupation**																								
Workers	1.06	(0.92)	-	(1.23)	1.00	(0.86)	-	(1.18)	0.66	(0.47)	-	(0.93)	0.96	(0.87)	-	(1.06)	1.02	(0.92)	-	(1.14)	0.90	(0.74)	-	(1.09)
Non-workers	1.00	-		-	1.00	-		-	1.00	-		-	1.00	-		-	1.00	-		-	1.00	-		-
**Smoking**																								
Yes	1.00	-		-	1.00	-		-	1.00	-		-	1.00	-		-	1.00	-		-	1.00	-		-
No	1.22	(1.07)	-	(1.38)	1.29	(1.12)	-	(1.48)	0.74	(0.54)	-	(1.02)	0.89	(0.69)	-	(1.16)	1.11	(0.82)	-	(1.52)	0.56	(0.37)	-	(0.86)
**Alcohol consumption**																								
Yes	1.05	(0.93)	-	(1.19)	1.10	(0.96)	-	(1.27)	0.84	(0.62)	-	(1.15)	0.99	(0.89)	-	(1.09)	0.84	(0.75)	-	(0.93)	0.63	(0.52)	-	(0.77)
No	1.00	-		-	1.00	-		-	1.00	-		-	1.00	-		-	1.00	-		-	1.00	-		-
**Daily energy intake**																								
Less than recommended energy intake per day	1.00	-		-	1.00	-		-	1.00	-		-	1.00	-		-	1.00	-		-	1.00	-		-
Recommended energy intake per day	0.94	(0.81)	-	(1.10)	1.02	(0.86)	-	(1.21)	0.92	(0.62)	-	(1.36)	0.93	(0.82)	-	(1.04)	0.89	(0.78)	-	(1.01)	0.97	(0.76)	-	(1.22)
More than recommended energy intake per day	1.06	(0.93)	-	(1.20)	1.04	(0.91)	-	(1.20)	0.72	(0.51)	-	(1.03)	1.05	(0.94)	-	(1.17)	1.03	(0.91)	-	(1.16)	1.01	(0.81)	-	(1.25)
**Physical activity**																								
Yes	1.01	(0.90)	-	(1.13)	1.04	(0.92)	-	(1.18)	0.67	(0.49)	-	(0.91)	0.96	(0.88)	-	(1.06)	0.99	(0.89)	-	(1.09)	0.77	(0.64)	-	(0.93)
No	1.00	-		-	1.00	-		-	1.00	-		-	1.00	-		-	1.00	-		-	1.00	-		-
**Diabetes mellitus**																								
No	1.00	-		-	1.00	-		-	1.00	-		-	1.00	-		-	1.00	-		-	1.00	-		-
Yes	1.53	(1.27)	-	(1.84)	1.20	(0.98)	-	(1.48)	0.44	(0.24)	-	(0.81)	1.65	(1.39)	-	(1.96)	1.23	(1.01)	-	(1.50)	0.47	(0.24)	-	(0.91)

**Table 3:** Factors associated with BMI according to the daily eating-out rate.

Variable	Obesity									Overweight									Underweight								
	None	1%–50%				51%–100%				None	1%–50%				51%–100%				None	1%–50%				51%–100%			
	OR	OR	95% CI			OR	95% CI			OR	OR	95% CI			OR	95% CI			OR	OR	95% CI			OR	95% CI		
**Men**																											
**Marital status**																											
Unmarried	1	0.29	(0.09)	-	(0.91)	0.32	(0.10)	-	(1.02)	1	0.53	(0.14)	-	(1.97)	0.61	(0.16)	-	(2.31)	1	0.09	(0.02)	-	(0.44)	0.11	(0.02)	-	(0.52)
Married	1	1.16	(0.89	-	(1.51)	1.44	(1.06	-	(1.97)	1	1.05	(0.80)	-	(1.38)	1.19	(0.85)	-	(1.65)	1	0.62	(0.37)	-	1.03)	0.89	(0.44)	-	(1.79)
Once married (divorced, separated, bereavement)	1	1.2	(0.60)	-	(2.40)	0.8	(0.34)	-	(1.88)	1	0.79	(0.39)	-	(1.60)	0.8	(0.34)	-	(1.87)	1	0.85	(0.26)	-	(2.79)	0.98	(0.22)	-	(4.49)
**Household income**																											
Low	1	1.08	(0.77)	-	(1.52)	1.18	(0.70)	-	(1.99)	1	0.86	(0.60)	-	(1.21)	0.76	(0.42)	-	(1.35)	1	0.74	(0.41)	-	(1.34)	0.42	(0.13)	-	(1.33)
Medium-Low	1	1.1	(0.70)	-	(1.74)	1.24	(0.74)	-	(2.10)	1	1.87	(1.08)	-	(3.26)	1.63	(0.87)	-	(3.06)	1	0.23	(0.10)	-	(0.51)	0.48	(0.18)	-	(1.26)
Medium-High	1	1.17	(0.60)	-	(2.28)	1.42	(0.70)	-	(2.88)	1	0.91	(0.46)	-	(1.81)	1.14	(0.55)	-	(2.38)	1	0.43	(0.11)	-	(1.67)	0.64	(0.15)	-	(2.75)
High	1	0.9	(0.36)	-	(2.25)	1.01	(0.39)	-	(2.60)	1	0.68	(0.28)	-	(1.64)	0.87	(0.35)	-	(2.14)	1	>999.999	<0.001	-	>999.999	>999.999	<0.001	-	>999.999
**Educational level**																											
Elementary school	1	1.12	(0.80)	-	(1.59)	0.92	(0.52)	-	(1.63)	1	1.06	(0.74)	-	(1.50)	1.03	(0.58)	-	(1.84)	1	0.82	(0.46)	-	(1.49)	0.36	(0.10)	-	(1.32)
Middle school	1	1.04	(0.58)	-	(1.85)	0.95	(0.46)	-	(1.98)	1	0.9	(0.49)	-	(1.63)	0.85	(0.39)	-	(1.84)	1	0.41	(0.14)	-	(1.16)	0.51	(0.12)	-	(2.25)
High school	1	1.27	(0.75)	-	(2.15)	1.49	(0.85)	-	(2.61)	1	0.98	(0.56)	-	(1.70)	1.1	(0.61)	-	(2.00)	1	0.29	(0.11)	-	(0.79)	0.32	(0.11)	-	(0.97)
College or more	1	0.81	(0.35)	-	(1.88)	1.03	(0.44)	-	(2.44)	1	0.71	(0.30)	-	(1.69)	0.85	(0.35)	-	(2.09)	1	0.29	(0.06)	-	(1.49)	0.81	(0.15)	-	(4.54)
**Occupation**																											
Workers	1	1.1	(0.77)	-	(1.58)	1.29	(0.88)	-	(1.90)	1	1.09	(0.74)	-	(1.60)	1.17	(0.77)	-	(1.78)	1	0.4	(0.19)	-	(0.84)	0.51	(0.22)	-	(1.18)
Non-workers	1	1.07	(0.77)	-	(1.48)	1.17	(0.76)	-	(1.82)	1	0.86	(0.62)	-	(1.20)	1.19	(0.75)	-	(1.90)	1	0.62	(0.36)	-	(1.07)	0.92	(0.42)	-	(2.02)
**Women**																											
**Marital status**																											
Unmarried	1	1.09	(0.38)	-	(3.13)	1.19	(0.41)	-	(3.50)	1	2.37	(0.42)	-	(13.23)	2.93	(0.52)	-	(16.56)	1	2.23	(0.36)	-	(14.00)	1.92	(0.30)	-	(12.19)
Married	1	1.3	(1.05)	-	(1.62)	1.56	(1.21	-	(2.01)	1	1.37	(1.08)	-	(1.74)	1.5	(1.13)	-	(2.00)	1	0.86	(0.47)	-	(1.56)	0.92	(0.48)	-	(1.77)
Once married (divorced, separated, bereavement)	1	1.24	(0.96)	-	(1.61)	1.41	(0.96)	-	(2.06)	1	1.31	(0.97)	-	(1.75)	1.31	(0.85)	-	(2.00)	1	0.66	(0.36)	-	(1.22)	0.43	(0.15)	-	(1.19)
**Household income**																											
Low	1	1.34	(1.06)	-	(1.69)	1.49	(1.01)	-	(2.19)	1	1.34	(1.03)	-	(1.74)	1.12	(0.71)	-	(1.76)	1	0.77	(0.43)	-	(1.39)	0.89	(0.38)	-	(2.10)
Medium-Low	1	1.13	(0.83)	-	(1.54)	1.43	(0.99)	-	(2.08)	1	1.5	(1.05)	-	(2.16)	1.52	(0.98)	-	(2.35)	1	0.93	(0.41)	-	(2.10)	1.2	(0.50)	-	(2.89)
Medium-High	1	1.3	(0.85)	-	(1.98)	1.41	(0.89)	-	(2.24)	1	1.37	(0.84)	-	(2.24)	1.79	(1.05)	-	(3.05)	1	0.57	(0.22)	-	(1.53)	0.48	(0.17)	-	(1.34)
High	1	1.24	(0.66)	-	(2.30)	1.42	(0.73)	-	(2.75)	1	1.08	(0.57)	-	(2.04)	1.2	(0.61)	-	(2.35)	1	0.93	(0.24)	-	(3.61)	0.78	(0.19)	-	(3.15)
**Educational level**																											
Elementary school	1	1.27	(1.04)	-	(1.54)	1.4	(0.96	-	2.03)	1	1.3	(1.04)	-	(1.63)	1.42	(0.94)	-	(2.17)	1	0.62	(0.37	-	1.06)	0.43	(0.12)	-	(1.60)
Middle school	1	1.35	(0.86)	-	(2.11)	1.61	(0.92	-	2.82)	1	1.62	(0.97)	-	(2.72)	1.58	(0.83)	-	(3.04)	1	4.14	(0.48	-	35.74)	1.02	(0.07)	-	(15.57)
High school	1	0.95	(0.61)	-	(1.49)	1.07	(0.67	-	1.72)	1	1.53	(0.88)	-	(2.65)	1.59	(0.89)	-	(2.83)	1	0.8	(0.30	-	2.12)	0.87	(0.32)	-	(2.37)
College or more	1	1.06	(0.48)	-	(2.32)	1.28	(0.57	-	2.85)	1	1.13	(0.47)	-	(2.75)	1.37	(0.56)	-	(3.39)	1	1.83	(0.41	-	8.21)	1.67	(0.37)	-	(7.61)
**Occupation**																											
Workers	1	1.38	(1.05)	-	(1.82)	1.44	(1.06	-	1.98)	1	1.19	(0.88)	-	(1.61)	1.29	(0.92)	-	(1.81)	1	0.7	(0.35	-	1.40)	0.65	(0.31)	-	(1.35)
Non-workers	1	1.24	(1.01)	-	(1.51)	1.67	(1.28	-	2.19)	1	1.48	(1.17)	-	(1.87)	1.64	(1.21)	-	(2.23)	1	0.87	(0.53	-	1.41)	0.9	(0.51)	-	(1.59)
